# Prevalence of Chronic Complications, Their Risk Factors, and the Cardiovascular Risk Factors among Patients with Type 2 Diabetes Attending the Diabetic Clinic at a Tertiary Care Hospital in Sri Lanka

**DOI:** 10.1155/2018/4504287

**Published:** 2018-05-23

**Authors:** Maulee Hiromi Arambewela, Noel P. Somasundaram, Hettiarachchige Buddhi Pradeep Ranjan Jayasekara, Mahesh P. Kumbukage, Pulukkutti Mudiyanselage Sarath Jayasena, Chandrasekara Mudalige Priyanka Hemanthi Chandrasekara, Kurukulasuriya Ravindra Alexis Sudath Fernando, Divadalage Priyantha Kusumsiri

**Affiliations:** ^1^Department of Diabetes and Endocrinology, National Hospital of Sri Lanka, Colombo, Sri Lanka; ^2^Faculty of Medical Sciences, University of Sri Jayewardenepura, Nugegoda, Sri Lanka; ^3^Ministry of Health, Colombo, Sri Lanka

## Abstract

Diabetes incurs heavy burden to patients and the healthcare system. Assessment of disease burden is important in taking necessary precautions and management decisions. We aimed to determine the prevalence of macro- and microvascular complications, their risk factors, and coronary artery disease (CAD) risk factors among patients with type 2 diabetes mellitus (T2DM). A descriptive cross-sectional single-centre study was carried out among 3000 patients with T2DM attending the diabetic clinic at the National Hospital of Sri Lanka from January to July 2016. The study population had 72.7% females and 27.3% males. Mean age and disease duration were 58.3 ± 10.3 and 10.8 ± 7 years, respectively. Prevalence of CAD, stroke, and peripheral vascular disease were 10.6%, 1.1%, and 4.7% while diabetic retinopathy, neuropathy, nephropathy, diabetic foot, and lower extremity amputation (LEA) were 26.1%, 62.6%, 50.8%, 2.6%, and 1.3%, respectively. Prevalence of overweight/obesity, hypertension, dyslipidemia, and smoking were 80%, 77.6%, 76.7%, and 11%, respectively. Increased age, disease duration, and HBA1c were risk factors for microvascular disease and diabetic foot while age was the only risk factor for macrovascular complications. Occurrence of CAD, peripheral neuropathy, diabetic foot, and LEA was significantly higher among males than when compared to females. This study highlights the major burden of chronic complications and high prevalence of CAD risk factors in this population.

## 1. Introduction

The global epidemic of diabetes has become one of the biggest challenges to mankind in the 21st century. The International Diabetes Federation in a recent report estimated that in 2011, 366 million people worldwide had diabetes, and if the trend continues by 2030, 552 million people or one in ten adults will suffer from diabetes [1]. The Western Pacific region was shown to have the largest number of people with diabetes (132 million) followed by the Southeast Asian region (71.4 million) [1]. The unprecedented rise in diabetes in the South Asian region has had its toll in Sri Lanka as well. According to statistics in 2006, one in five adults in Sri Lanka was diabetic or prediabetic with one-third of those with diabetes being undiagnosed [2]. South Asians are at risk of increased visceral adiposity, insulin resistance, and impaired *β* cell function and are genetically predisposed to diabetes [3–5]. Additionally, economic development following the ending of the 30-year-old war in Sri Lanka has led to rapid urbanization causing decreased physical activity and consumption of fast food causing amplification of this risk and increasing the incidence of this disease.

Chronic hyperglycemia results in multisystemic complications of the eyes, nerves, kidneys, heart, and blood vessels. The disease burden of diabetes is mainly attributed to the morbidity and mortality associated with microvascular and macrovascular complications. The UK prospective study done among patients with type 2 diabetes showed that intensive glycemic control reduces the risk of development of micro- and macrovascular complications [6]. However, a significant number of patients harbor these complications as well as other metabolic risk factors even prior to the diagnosis of diabetes [7]. Newly diagnosed South Asians with diabetes have a higher prevalence of these vascular complications at the time of diagnosis when compared to European macrovascular complications (15.7% versus 9.4%) and microvascular complications (27.3% versus 16.5%) [8]. Poor glycemic control and duration of diabetes seem to be the strongest risk factors for the development of vascular complications while other factors such as hypertension, dyslipidemia, obesity, smoking, age, and genetic factors all contribute. It is also notable that the incidence of CAD among South Asians is higher when compared to that among Europeans [9]. In a study done among diabetic patients in the United Kingdom (UK), South Asians were 3.8 times more likely to develop myocardial infarction [10]. In contrast, the UK prospective study 32 did not detect a significant difference in the rates of CAD among South Asians and white Europeans [11]. However, according to the study authors, this finding could be due to potential biases. In another large study done in Northern California, there was similar incidence between South Asians and Whites but much higher than the other ethnic groups [12]. Larger and more long-term studies are required to determine if the actual CAD risk is higher among South Asians. Several other studies have demonstrated that South Asians with diabetes have a higher mortality rate of CAD when compared to other ethnic groups [10, 13–15]. However, on the other hand, a large study done in Canada reported that South Asians had a lower mortality rate compared to their Canadian counterparts [16]. In a large multinational study on the prevalence of diabetes complications by Litwak et al., macrovascular complications were reported in 23.3% and microvascular complications in 39% among the patients in South Asia [17]. Surprisingly, Russia had the highest prevalence of 72% for macrovascular and 89% for microvascular complications. European and North American countries were not taken into consideration in this study. A large study done in Sri Lanka in 2012 among patients with diabetes attending an outpatient clinic in the Western Province reported prevalence of CAD (5.6%), peripheral vascular disease (PVD) (0.5%), neuropathy (28.4%), nephropathy (20.4%), and retinopathy (25.7%) [14]. Another small study done in Sri Lanka among 147 inward patients in 2012 reported prevalence of CAD, stroke, PVD in 52.6%, 6.2%, and 4.1% and neuropathy, nephropathy, and retinopathy in 31%, 19%, and 28.7%, respectively [18]. However, the difference in prevalence could be due to different patient populations and sample sizes. Patients who are inward are probably more likely to have a higher disease burden from chronic complications. In another study done in Jaffna (Northern Province) among 8400 outpatients, CAD was present in 21.1%, stroke in 3.9%, peripheral neuropathy in 34.1%, and nephropathy in 39.5% [19]. In a study done in the Eastern Province, the prevalence for heart disease, renal impairment, and eye disease among patients with diabetes was 22.5%, 22.5%, and 56%, respectively [20]. However, the variables have not been clearly defined in this study.

Micro- and macrovascular complications cause many disabilities to the patient leading to reduction of quality of life and incur a heavy burden on the free healthcare system. Diabetes is a major global cause of premature mortality that is widely underestimated. CAD is the biggest killer and is the cause for mortality in more than 70% of diabetics [21]. This is the reason for the change in focus from managing only hyperglycemia to managing hyperglycemia along with the other cardiovascular risk factors. It is apparent that evidence on the prevalence of diabetes-related complications is essential for the adjustment of policies and practices in diabetic care management. Screening for macro- and microvascular complications will have important implications for understanding the need of vigorous screening and for planning out effective preventive and management strategies in order to lessen the burden of this chronic debilitating disease on healthcare resources and expenditure. Therefore, it is important that such prevalence studies are done from time to time to detect the changing trends in order to plan out the course of action. Even though many such studies have been done in different parts of the country, a comprehensive study has not been done recently among the outpatients in the diabetic clinic at the National Hospital of Sri Lanka, which caters to a large patient population in the capital city and its suburban areas. Therefore, the aim of this study was to assess the prevalence of macro- and microvascular complications and their association with possible risk factors and to describe the cardiovascular risk factors in a large cohort of patients with type 2 diabetes.

## 2. Methods

This was a descriptive cross-sectional single-centre study carried out at the National Hospital of Sri Lanka during the time period of 1 January 2016 to 31 July 2016. The National Hospital is the biggest hospital in Sri Lanka with a bed strength of more than 3000. Around 16,000 patients are registered in the diabetes clinic, and around 400 patients a day attend the clinic to seek care. It is a tertiary care referral centre and caters mainly to diabetic patients in Colombo and its suburbs. A total of 3000 patients with type 2 diabetes (T2DM) were systematically sampled. Pregnant patients, patients with gestational diabetes, and patients with type 1 diabetes were excluded. All patients included in the study were screened for vascular complications.

Sociodemographic data was recorded by specially trained data collectors. Height, weight, and blood pressure were measured and recorded by trained health staff. Screening for diabetic complications and extracting disease-related data from the health records were performed by specially trained doctors in the diabetes clinic.

### 2.1. Baseline Data Definitions

#### 2.1.1. Type 2 Diabetes

Patients were diagnosed with diabetes according to the American Diabetes Association criteria of fasting blood glucose of over 126 mg/dl or a value of >200 mg/dl in the 2 hr value in the oral glucose tolerance test. Two values were used if the patient was asymptomatic, and one value was used if the patient was symptomatic for diagnosis. Patients who had been diagnosed for at least 3 months were included. Patients with other forms of diabetes such as type 1 diabetes, maturity-onset diabetes (MODY), and latent autoimmune diabetes of adults (LADA) were excluded if they already carried such a diagnosis or on clinical criteria.

#### 2.1.2. Complications

Presence of ischemic heart disease and stroke/transient ischemic attack was considered if the patient had such evidence in the medical records. Peripheral vascular disease was considered if the ankle brachial pressure index was less than 0.9.

Neuropathy was diagnosed if either touch sensation detection using 10 g monofilament test showed a score of less than 7 out of 10 or vibration sensation detection using biothesiometer was abnormal. Retinopathy was diagnosed by detailed fundus examination by retinoscope and classified into diabetic maculopathy, nonproliferative retinopathy, and proliferative retinopathy.

Nephropathy was diagnosed based on the urine albumin/creatinine ratio (UACR) of a single urine spot sample. A ratio of >2.5 mg/mol in males and >3.5 mg/mol in females was considered abnormal.

Diabetic foot disease was defined if the patient had an amputation or current or past history of foot ulcer which took 2 weeks or more to heal in the presence of peripheral vascular disease and peripheral neuropathy.

Staging of chronic kidney disease was calculated by using the Cockcroft-Gault equation.

#### 2.1.3. Ethical Issues

Ethical clearance was obtained from the ethical review committee of the University of Colombo prior to the initiation of the study. Administrative approval was obtained from the hospital representatives. Participation was entirely voluntary and written informed consent was obtained from the participants. The patients had the full liberty to exit the study at any given point of time if they wished to do so.

#### 2.1.4. Statistical Analysis

Statistical analysis was performed using the Statistical Package for Social Sciences (SPSS) version 20. Data was reported as mean ± SD and percentages. Differences of means between males and females were calculated using independent sample *t*-test for skewed variables with Wilcoxon rank test. Categorical variables were analysed by using chi-square and Fisher's exact tests. Significant level was set at 5%. Results were expressed as odds ratios (OR) and 95% confidence intervals (CI). Associations for predictor variables with each chronic complications were examined initially with bivariate analysis and then with binary logistic regression separately. Backward stepwise method was used to do the regression modeling. The main model consists of the following variables: duration of the illness, HBA1C level, fasting blood sugar values (FBS), postprandial blood sugar levels (PPBS), systolic blood pressure (SBP), diastolic blood pressure (DBP), body mass index (BMI), and gender.

## 3. Results

**Figure 1:**
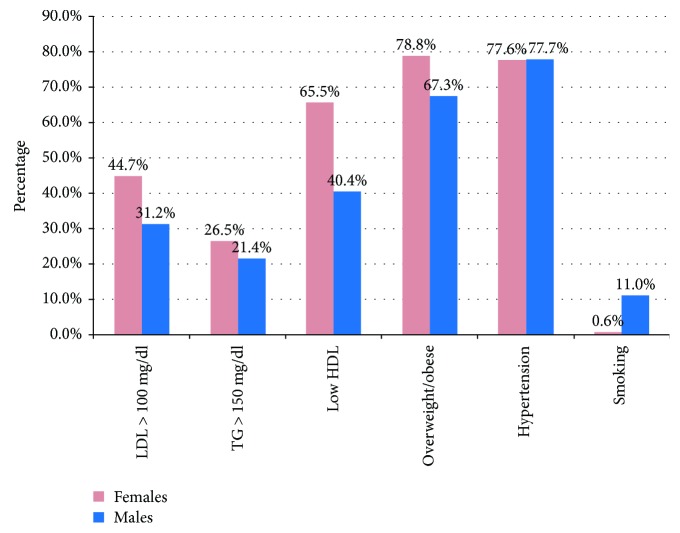
Prevalence of cardiovascular risk factors in the study population by gender (females *n* = 2180, males *n* = 820).

Out of the 3000 patients studied, 2180 were females (72.7%) and 820 (27.3%) were males. Mean age ± SD was 58.3 ± 10.3 years with 68.3% of the total population falling within the age group of 50–70. Only 4.5% of patients were below the age of 40 years. Mean duration of diabetes ± SD was 10.8 ± 7.3 years with 42.9% of patients having disease duration of over 10 years. The metabolic profile of the study population is given in Table 1. It is also noteworthy that 75.7% of the total population were either overweight or obese with a basal metabolic index (BMI) of >23. Females had a higher BMI compared to males (26.5 versus 24.8). However, this difference was not significant. All parameters of glycemic, blood pressure, and lipid control were higher in females than in males but the observed difference was significant only for HBA1c (*p* = 0.04), fasting blood sugar (FBS) (*p* = 0.005), and systolic blood pressure (SBP) (*p* < 0.001).  Figure 1 illustrates the cardiovascular risk factors in the study population by gender. This reiterates again the pressing issue of obesity with more than three-fourths of the population falling into the overweight/obese category. Low high-density lipoprotein cholesterol (HDL) levels were seen among 65.5% of the females and 40.4% of the males. High low-density lipoprotein (LDL) cholesterol levels were seen in 41% of the population, and 25% had high triglyceride levels. These lipid derangements were in spite of 88.6% of the patients who had been on statin therapy. The prevalence of hypertension in the study population was 77.6% with no significant difference between the genders. However, blood pressure was not under control in 50.3% of the population in spite of treatment. The prescription of angiotensin-converting enzyme inhibitors (ACEI)/angiotensin receptor blockers (ARB), calcium channel blockers (CCB), thiazide diuretics, beta blockers, alpha blockers, and methyldopa was 70%, 15.9%, 16.5%, 15.3%, 5.2%, and 0.1%, respectively. Among the males in the study population, 11% were current smokers while 48.4% were ex-smokers, and 40.6% said they have never smoked before. Almost all the females (98.4%) said they have never smoked before.

The prevalence of vascular complications is described in Table 2. CAD was seen among 10.5% of the population with 0.6% and 1.9% having undergone angioplasty and coronary artery bypass grafting, respectively. Stroke and peripheral vascular disease was seen in 1.1% and 4.7% of the total population, respectively. The prevalence of diabetic retinopathy was 26.1% in the total study population with nonproliferative retinopathy, proliferative retinopathy, and diabetic maculopathy seen in 21.3%, 1.6%, and 6.2%, respectively. Neuropathy, nephropathy, and diabetic foot were seen in 62.6%, 50.8%, and 2.6%, respectively. Out of the total patient population, 41.1% had chronic kidney disease (CKD) staging of 2, and 27.1% had a CKD staging of 3. Only 3.2% of patients belonged to CKD stages of 4 and 5. In the study sample, 13.7% had at least one macrovascular complication, and 74% had at least one microvascular complication while 10.5% had both macro- and microvascular complications. All vascular complications except peripheral vascular disease were more prevalent among males than females. However, statistically significant difference was observed with CAD (*p* = 0.007), neuropathy (*p* = 0.001), diabetic foot (*p* = 0.001), and lower extremity amputation (LEA) (*p* = 0.001) only.

**Figure 2:**
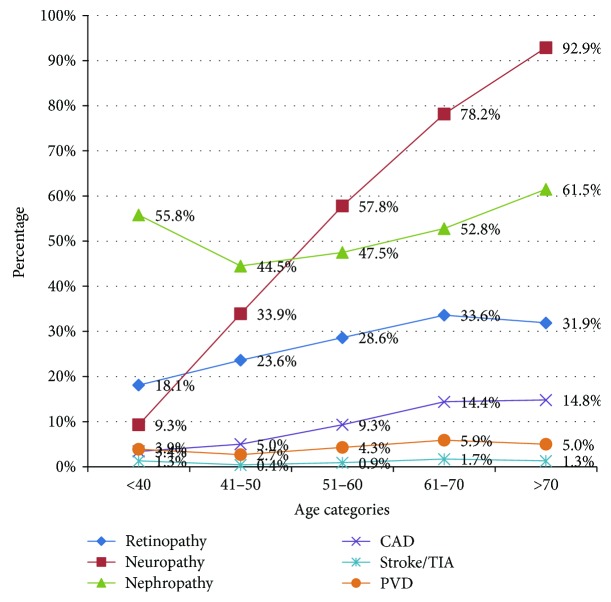
Variation of chronic complications with age among 3000 patients with type 2 diabetes.

**Figure 3:**
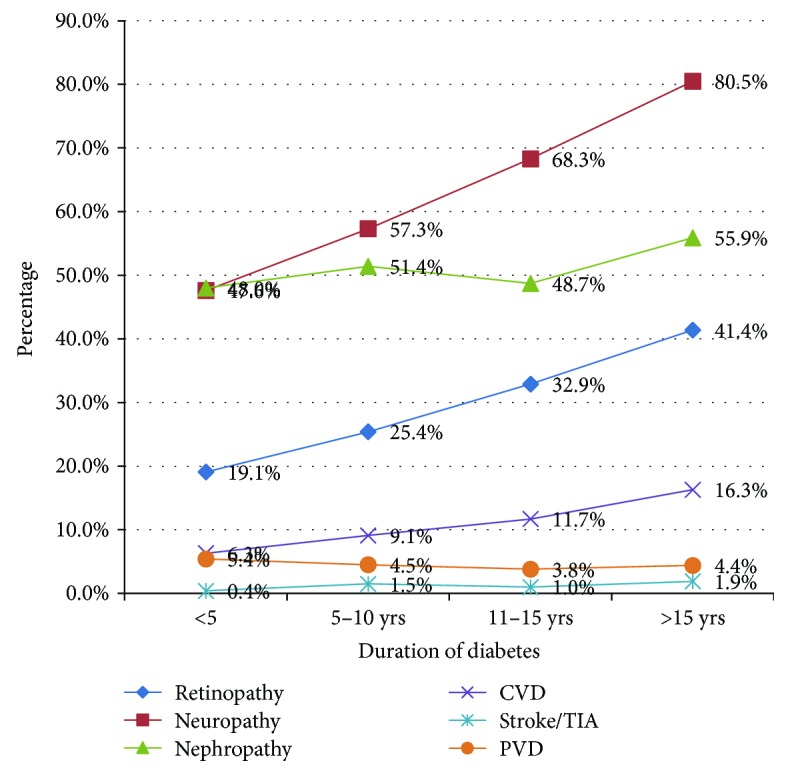
Variation of chronic complications with disease duration among 3000 patients with type 2 diabetes.

The variation of chronic complications with age of the patient and duration of diabetes is demonstrated in Figures 2 and 3, respectively. According to these graphs, it is evident that all the chronic complications in diabetes increase with age and the duration of the illness. Out of all the complications, neuropathy is the commonest complication followed by nephropathy and retinopathy. Among the patients who had diabetes for more than 15 years, 80.5% were having neuropathy while retinopathy and nephropathy were seen among 55.9% and 41.4%, respectively. Logistic regression analysis was conducted with stepwise method using backward selection. As shown in Table 3 microvascular complications were significantly associated with age > 60 years (OR 1.92, 95% CI 1.41–2.73; *p* < 0.001), duration of diabetes > 10 years (OR 2.14, 95% CI 1.58–2.88; *p* < 0.001), and HBA1c > 7% (OR 1.37, 95% CI 1.01–1.87; *p* < 0.04) while macrovascular complications were associated with age > 60 years (OR 1.82, 95% CI 1.36–2.44; *p* < 0.001) only.

A comparison of vascular complications with other regions is demonstrated in Table 4. Accordingly, macrovascular complications are lower and microvascular complications higher than the other regions in Asia.

## 4. Discussion

Sri Lanka is currently witnessing an unprecedented rise in the prevalence of diabetes mainly due to the cultural, demographical, behavioral, and environmental changes brought on by rapid urbanization and globalization. Abdel Omran's theory on the “epidemiological transition” where victory over infectious disease is allowing people to live longer and hence develop chronic noncommunicable disease perhaps may be another contributory factor [23]. According to the WHO 2014 update, the three highest causes of death in Sri Lanka were reported as CVD, stroke, and diabetes. A study done on trends in Sri Lankan cause-specific adult mortality concluded that chronic diseases are the greatest cause of morbidity and mortality and highlighted the importance in adaptation of the health sector to this alarming trend [22].

This study was carried out at the National Hospital in Colombo, which is the largest hospital and referral centre in Sri Lanka, catering to diabetic patients in Colombo and its suburbs as well as the complex diabetic patients referred by other units in the hospital. The prevalence of chronic complications was high among these patients with 84.4% suffering from at least one vascular complication. Retinopathy was seen in more than one-fourth of the population with sight-threatening retinopathy in 8%. Nephropathy was seen in half of the patient population. However, nephropathy in this setting was defined based on a single UACR. According to the 2017 “Standards of medical care in diabetes” by the American Diabetes Association (ADA), two out of three reports done within 3–6 months should be positive to de diagnosed with nephropathy leaving allowance for biological variability. Due to the limited availability of this test, we had to rely on a single value, and thus the actual percentage of patients with nephropathy would have been lower than this.

According to Table 4, the prevalence of macrovascular complications seems to be lower, and microvascular complications seem to be higher in Sri Lanka, when compared to other Asian countries. However, this data was from a multinational study carried out in 2009-2010, and the current prevalence may be different [17]. Compared to Sri Lankan data from a study done in 2012 at the National Diabetic Centre in Colombo, both macro- and microvascular disease burdens have increased, with the prevalence of CVD almost double the prevalence reported in 2012 [14]. The National Diabetic Centre is a nonprofitable organization which caters to patients with diabetes in Colombo and from the suburban areas. The present study was carried out among the outpatients in the largest tertiary care hospital in Colombo. The increase in disease burden over the years could actually be due to the rising trend of chronic complications. However, this would have been further compounded by the concentration of patients with complex disease in this tertiary care referral centre. Differences in defining various vascular complications would also have contributed to this discrepancy. A higher prevalence of vascular complications among a small sample of inward patients with diabetes in the same tertiary care hospital was reported by Perera et al. [18]. However, this is probably due to higher disease burden seen among patients with diabetes who require admission. It is also interesting to note that two recent studies done in the Northern and Eastern Provinces of Sri Lanka reported a much higher prevalence of CAD [19, 20]. Future research may be warranted to determine if there is a significant difference in prevalence of CAD among various provinces and of any possible contributory risk factors.

Determining the risk factors for the development of micro- and macrovascular angiopathy is important in order to attempt to reduce the burden from disease complications. Previous studies have shown that age, duration of diabetes, and age at diagnosis have varying effects on the risk of angiopathy. The UK Prospective Diabetes Study (UKPDS) reported an increase in the prevalence of myocardial infarction but not in retinopathy or nephropathy with old age in patients newly diagnosed with diabetes. In contrast, after follow-up of 6 years, younger age at diagnosis was associated with an increased risk of retinopathy and nephropathy but not so in risk of myocardial infarction or nephropathy [24]. This suggests that interrelation between age, age at diagnosis, and duration of diabetes is complex with regard to angiopathy in different organ systems and further complicated by the unquantified time period of hyperglycemia prior to diagnosis. A general inference is that macrovascular events are more common among the elderly even among nondiabetics, and this is further exacerbated with the setting in of diabetes. Microvascular complications however are more closely related to diabetes, and the risk of these is closely related to disease duration [25]. In this study, microvascular complications were significantly associated with age, disease duration, and glycemic control while the only factor significantly associated with macrovascular complications was age. Many prospective randomized controlled trials have shown that persistent intensive glycemic control reduces the risk of mainly microangiopathy as well as macroangiopathy in the long term. In our study, poor glycemic control was significantly associated with microvascular disease but not so with macrovascular disease. Diabetic foot is a complication which arises due to combination of macro- and microangiopathy in the background of poor glycemic control. According to our findings, age, duration of diabetes, and poor glycemic control were all associated with diabetic foot. Previous studies have also shown that hypertension and smoking play a role in microangiopathy [26, 27]. In this study, high systolic blood pressure was associated only with retinopathy, and none of the chronic complications were associated with smoking. It is important to keep in mind that this study was a descriptive cross-sectional study looking at data at a given point of time. The effect of these risk factors on vascular complications cannot be determined by such a methodology and would require long-term prospective randomized control trials.

Abundant evidence shows that diabetes is a risk factor for CAD, and it is now the leading cause of diabetes-related morbidity and mortality. Patients with T2DM with insulin resistance have a proatherogenic cardiovascular risk profile, which includes risk factors such as poor glycemic control, hypertension, abdominal obesity, microalbuminuria, smoking, and atherogenic lipid profile with reduced LDL cholesterol, increased TG, and reduced HDL cholesterol levels. However, we were unable to find any significant association between these risk factors and the occurrence of CAD in this population. This is not unusual as such variations have been reported previously [27, 28]. The descriptive cross-sectional nature of the study design would have also contributed to this as we have looked at these risk factors at only one given point of time. Nevertheless, it is important to note that the prevalence of these well-documented CVD risk factors in this population is high (Figure 1).

Obesity seems to be a major problem with almost 75.7% of the patients with diabetes being overweight or obese. Most of these patients are invariably suffering from metabolic syndrome. Obesity is an emerging global problem giving rise to increase in diabetes, hypertension, dyslipidemia, metabolic syndrome, CVD, stroke, cancer, and reproductive and psychosocial diseases. Prospective studies which have followed up patients for more than two decades have documented that obesity is an independent risk factor for CVD [29]. However, it is a heterogenous condition, and it is mainly the abdominal obesity that is associated with excess cardiovascular risk [30]. Research evaluating the association with BMI and risk of death among patients with diabetes has shown inconsistent results with many studies showing a U-shaped association with BMI and all-cause mortality [31]. Katulanda et al. reported a high prevalence of overweight (25.2%), obese (9.2%), and centrally obese (26.2%) among the general population of Sri Lanka [32]. The prevalence of overweight and obese individuals in our study population was alarmingly high at 75.7%. However, central obesity was not measured in this study.

Hypertension in diabetics is an important issue as the combination often coexists. It affects around 30% of the European diabetic population [33]. Several studies have shown the close association of diabetes with hypertension. Hypertension is significantly more prevalent among patients with type 2 diabetes. This link is mainly attributed to hyperinsulinemia [34]. The prevalence of hypertension is 1.5 to 2 times more in patients with diabetes than in those without diabetes, while almost one-third of patients with hypertension develop diabetes later [35]. The presence of hypertension will increase the risk of CAD, stroke, retinopathy, and nephropathy. In this study, the prevalence of hypertension was 77.6%. This was significantly higher than neighboring India where a study reported hypertension among only 25% of the patients with T2DM [36]. The American Diabetes Association recommends patients with diabetes to achieve a blood pressure goal of less than 140/90 mmHg [37]. Treatment of hypertension in these patients should include an agent which has shown to reduce cardiovascular events (ACEI/ARB, thiazide-like diuretics, and dihydropyridine CCB). Multiple drugs are usually needed to achieve blood pressure targets. In our study, 70% of patients were on ACEI/ARB followed by 16.5% on thiazide-like diuretics and 15.9% on CCB. The superiority of ACE/ARBs over other antihypertensive agents for the prevention of CAD has not been consistently proven [38, 39]. In a recent meta-analysis, thiazide-like diuretics or dihydropyridine CCB have shown cardiovascular benefits similar to ACEI/ARB among patients without albuminuria [38]. It is important to note that blood pressure was inadequately controlled in almost half of the patients on treatment for hypertension in this study. More focus should be upon achieving targets using multiple drug combinations to alleviate the risk CAD.

Dyslipidemia is one of the key risk factors for CVD among diabetic patients. The characteristic features in diabetic dyslipidemia is a high TG and low HDL concentrations. Low LDL levels may not be significantly different from LDL levels among nondiabetics. However, all these promote atherogenesis. In this study population, the prevalence of dyslipidemia was 76.7%, with high LDL, high TG, and low HDL seen in 41%, 25%, and 54.7%, respectively. Out of the patients with dyslipidemia, 88.6% of the patients were on statin therapy. The most commonly deranged component of the lipid profile was HDL. Low HDL was commoner among females (61.2%) than among males (37.4%). Such gender-based predisposition in low HDL has been observed by Weerarathna et al. as well [40]. This may be due to the fact that most females in this study sample were menopausal and thus have lost the favorable increase in HDL due to estrogen. Furthermore, higher level of inactivity among this population may also contribute to the low HDL levels. As of present, guidelines do not recommend specific pharmacotherapy for increasing HDL apart from statins. According to the American Diabetes Association 2017 standards of care, men with TG levels of >204 mg/dl and HDL levels of <30 mg/dl who are already on statin can be commenced on a fibrate. High prevalence of dyslipidemia among these patients while on treatment suggests that intensive medical therapy and lifestyle modification need to be emphasized.

Among the males in the study population, 11% were current smokers. This is self rated, and more objective methods of assessing smoking would have been appropriate and yielded a higher prevalence. Smoking is an independent risk factor for all-cause mortality mainly due to CVD [41]. This should be reemphasized during the consultations with the patients.

It is interesting to note the gender-based discrepancy among the metabolic parameters and vascular complications in this study population. All metabolic parameters such as BMI, FBS, PPBS, HBA1c, blood pressure, LDL, and TG levels were higher in females than in men. However, statistical difference was observed for FBS, HBA1c, and systolic blood pressure. This was in contrast to vascular complications, where almost all the complications were more prevalent in men than in women with significant difference observed in CAD, neuropathy diabetic foot, and lower limb amputation. Data from the World Health Organization shows that men have twice as higher risk of developing CAD than women [42]. However, diabetes seems to erase this gender-based advantage that women have over men. Women with diabetes are said to have a high risk for CAD than men [43]. However, this fact is somewhat controversial. Some studies have shown that diabetes definitely eliminates this female advantage over men while other studies have not shown a significant difference [44, 45]. More studies would be required to determine the relationship between gender and CAD among patients with diabetes. Major complex gender differences exist between diabetes-related LEA. Evidence suggests that men are more liable to experience LEA than women [46, 47]. Men are more likely to have independent risk factors such as diabetic foot, PVD, cigarette use, and peripheral neuropathy leading to amputation [48, 49]. Sensory neuropathy is the most common neuropathy associated with amputation, and men have twice the risk of developing this than women. This gender-based predisposition was evident in our study population as well.

### 4.1. Limitations

There are several limitations of this study. The target population of patients attending the clinic in a tertiary referral centre reflects a population with more complex disease burden. Therefore, the prevalence reported may be an overestimation of the actual disease burden. Thus, to generalize the findings of this study to the entire population of patients with T2DM may not be quite appropriate. The diagnosis of CAD was based on medical records without considering if the patient had any current ischemic symptoms. The diagnosis of neuropathy was based only on the results of vibration and monofilament tests without taking the patients' symptoms into consideration. Due to the scarcity of laboratory resources, the diagnosis of nephropathy was based on a single UACR. Limiting the evaluation of obesity to general obesity without taking central obesity into consideration was another important limiting factor.

## 5. Conclusion

This study is evidence for the high prevalence of chronic vascular complications with increased disease burden. Age, duration of diabetes, and HBA1c were significantly associated with microvascular complications and diabetic foot while only age was associated with macrovascular complications. Furthermore, this population was at high risk of CVD with high prevalence of hypertension, dyslipidemia, obesity, and microalbuminuria. Men had a higher prevalence of CAD, peripheral neuropathy, diabetic foot, and LEA. Appropriate measures to intensify medical therapy and lifestyle measures to control modifiable risk factors and routine screening for the detection of new complications need to be emphasized in order to prevent morbidity and mortality.

## Figures and Tables

**Table 1:** Metabolic profile and its significance to the study population by gender.

Variable	Females (*n* = 2180) Mean ± (SD)	Males (*n* = 820) Mean ± (SD)	Total (*n* = 3000) Mean ± (SD)	*T* value	*p* value
Basal metabolic index (BMI)	26.5 ± (4.5)	24.8 ± (4.5)	26.3 ± (4.6)	1.06	0.289
HBA1c (%)	8.46 ± (1.8)	8.0 ± (3.7)	8.3 ± (2.5)	2.85	0.04
Fasting blood sugar (mg/dl)	159.6 ± (35.5)	127.9 ± (35.5)	137 ± (40.1)	2.80	0.005
Postprandial blood sugar (mg/dl)	162.6 ± (30.1)	156.0 ± (29.2)	161.1 ± (32.5)	0.92	0.35
Systolic blood pressure (mmHg)	131.5 ± (20.1)	128 ± (10.1)	130 ± (19.9)	3.92	0.001
Diastolic blood pressure (mmHg)	79.4 ± (10.2)	79.1 ± (11.8)	79.4 ± (10.7)	0.29	0.77
Low-density lipoprotein (mg/dl)	99.7 ± (9.1)	98.8 ± (18.7)	99.78 ± (14.10)	1.83	0.06
High-density lipoprotein (mg/dl)	47.8 ± (10.8)	45.8 ± (10.5)	46.93 ± (10.5)	1.35	0.17
Triglyceride (mg/dl)	127.8 ± (35.3)	118. 1 ± (29.5)	127.92 ± (35.0)	1.61	0.10

**Table 2:** Vascular complications by gender.

Disease complication	Overall prevalence *n* = 3000	Females *n* = 2180	Males *n* = 820	*X* ^2^ value	Significance
Cardiovascular diseases	318 (10.6%)	211 (9.7%)	107 (13.0%)	7.15	**0.007**
Stroke	33 (1.1%)	19 (0.8%)	14 (1.8%)	3.81	**0.05**
PVD	140 (4.7%)	105 (4.8%)	35 (4.2%)	0.81	0.36
Retinopathy	783 (26.1%)	545 (25%)	238 (29%)	2.62	0.10
Neuropathy	1879 (62.6%)	1332 (61.1%)	547 (68.5%)	20.3	**0.001**
Nephropathy	446/878 (50.8%)	306/631 (51.6%)	136/247 (55%)	2.77	0.96
Diabetic foot	78 (2.6%)	40 (1.8%)	38 (4.7%)	19.45	**0.001**
Lower extremity amputation	54 (1.3%)	26 (1.2%)	28 (3.4%)	15.09	**0.001**

**Table 3:** Logistic regression analysis showing risk factors which were significantly associated with chronic complications.

Complication	Risk factor	Odds ratio (95% confidence interval)	*p* value
Microvascular disease	Age (<60 yrs versus >60)	1.92 (1.41–2.73)	0.001
	Duration of diabetes (<10 yrs versus >10 yrs)	2.14 (1.58–2.88)	0.001
	HbA1c (<7% versus >7%)	1.37 (1.01–1.87)	0.04

Macrovascular disease	Age (<60 yrs versus >60)	1.82 (1.36–2.44)	0.001

Diabetic foot	Age (<60 yrs versus >60)	2.07 (1.13–3.80)	0.01
	Duration of diabetes (<10 yrs versus >10 yrs)	1.96 (1.05–3.65)	0.03
	HbA1c (<7% versus >7%)	2.20 (1.00–4.83)	0.04

**Table 4:** Comparison of chronic complications with other regions in Asia.

Complication	Sri Lanka 2016	Sri Lanka 2012	South Asia	East Asia	China	Middle East
Macrovascular complications	13.4%	—	23.3%	26.8%	21.3%	28.7%
CVD	10.5%	5.6%				
PVD	4.2%	0.5%				
Stroke	1.1%	—				
Microvascular complications	74%	—	39%	56%	49.6%	65.8%
Retinopathy	26.1%	25.7%	16.3%	23.7%	22.1%	33.9%
Nephropathy	50.8%	20.4%	20.3%	28.4%	22.3%	40.8%
Neuropathy	62.6%	28.4%	24.6%	36.95	33.3%	53.4%
Diabetic foot	2.6%	—	4.9%	5.3%	2.5%	8.6%

Data from other regions taken from a multinational study done from 2009-2010 [22].
